# Role of Trust, Risk Perception, and Perceived Benefit in COVID-19 Vaccination Intention of the Public

**DOI:** 10.3390/healthcare11182589

**Published:** 2023-09-20

**Authors:** Siu-Shing Man, Huiying Wen, Ligao Zhao, Billy Chun-Lung So

**Affiliations:** 1 School of Design, South China University of Technology, Guangzhou 510641, China; ssman6@scut.edu.cn (S.-S.M.); wenhuiying13@163.com (H.W.); 2 Guangzhou Huadu Huacheng Community Health Service Centre, Guangzhou 510810, China; zlgagain@163.com; 3 Department of Rehabilitation Sciences, The Hong Kong Polytechnic University, Hong Kong, China

**Keywords:** COVID-19 vaccination intention, perceived benefit, risk perception, theory of planned behavior, trust

## Abstract

COVID-19 vaccination is an effective method for dealing with the COVID-19 pandemic. This study proposed and validated a theoretical intention model for explaining the COVID-19 vaccination intention (CVI) of the public. The theoretical intention model incorporated trust in vaccines, two types of risk perception (risk perception of COVID-19 and risk perception of COVID-19 vaccination), and perceived benefit into a theory of planned behavior (TPB). Structural equation modeling was utilized to test the theoretical intention model with data collected from 816 Chinese adults in China. The results confirmed the crucial role of trust in vaccines, risk perception, and perceived benefit in shaping the CVI of the public. In addition, TPB was found to be applicable in a research context. The theoretical intention model accounted for 78.8% of the variance in CVI. Based on the findings, several practical recommendations for improving COVID-19 vaccination rates were discussed.

## 1. Introduction

The COVID-19 pandemic is a difficult challenge for every country. Compared with other infectious diseases, COVID-19 is more severe in terms of transmission speed, susceptibility, and mortality [1,2]. The World Health Organization is committed to calling on each country to diagnose and treat COVID-19 promptly and to actively facilitate the end of the COVID-19 pandemic [3]. Although the isolation, prevention, and control policies implemented by most countries and regions prevented most citizens from being infected, such policies cannot protect everyone from COVID-19, such as clinicians, the elderly, and people living in high-risk areas. The world can return to its normal state from before the COVID-19 pandemic by obtaining safe and effective vaccines and successfully implementing a global vaccination plan [4]. According to past infectious disease control experience, vaccination is an effective method for preventing the spread of infectious diseases and minimizing the occurrence of sequelae [5]. When vaccination rates are high, the infection curve of COVID-19 can be flattened [6]. However, recent public concern about the adverse effects of COVID-19 vaccines has caused people in the United States, the United Kingdom, and Australia to hesitate becoming vaccinated against the virus [7,8,9], which has directly affected vaccination rates. The vaccination rate is an important indicator reflecting the implementation of vaccination efforts [10]. People who are worried about the side effects of COVID-19 vaccines and have doubts about the development of the COVID-19 pandemic are reluctant to get vaccinated against the virus [11]. Vaccination rates vary widely across countries both at the start of campaigns and over time [12]. However, the effective rollout of a COVID-19 vaccine offers the most promising prospect for ending the pandemic [12]. Therefore, there is a need to promote vaccination and actively improve existing vaccination methods.

To increase the public’s COVID-19 vaccination intention (CVI), understanding the relevant factors affecting intention is critical [13]. Some excellent studies have been conducted in this research area. For example, cross-sectional studies have shown that public perception of COVID-19 vaccines, including their perceived threat and beliefs about their efficacy and safety, is related to whether individuals are willing to have their relatives and friends vaccinated [14]. People are also willing to receive COVID-19 vaccinations when they believe that the COVID-19 pandemic will not end soon [15]. Also, people’s beliefs and attitudes about the COVID-19 pandemic virus and vaccination against COVID-19 could explain 76% of the variance in vaccination intentions [16]. In this case, the risk to others that COVID-19 would cause and the need for vaccination could be emphasized. However, when people were subjected to scientific-sounding misinformation, their intention to vaccinate declined significantly [17]. Therefore, when conducting CVI-related research, it is necessary to pay attention to the channels through which people receive information and explore the extent to which different factors influence CVI. In addition, Giuliani et al. [18] investigated the relationship between vaccination intention and socio-demographic factors and showed that decreased CVI is related to age and occupation. Moreover, Drążkowski and Trepanowski [19] found that CVI is closely related to age, gender, perceived disease severity, and reactance, and Tam et al. [20] reported that college students consider authoritative advice from doctors and local availability of vaccines when deciding whether or not to get vaccinated against COVID-19. Meanwhile, Nguyen et al. [21] found that in addition to socio-demographic factors, residents’ trust in the government, concern about vaccine safety, and fear of rapid vaccine development were major factors affecting their vaccination intention. Therefore, increasing public confidence in vaccines, leading to a high level of vaccination intention, is essential to prevent further spread of COVID-19.

However, gaps exist in the research on the public’s CVI. First, a previous study by Goldman et al. [14] showed that individual willingness, such as perceived risk and public trust in vaccination, was related to the public’s CVI. However, the specific significance of the impact of such psychological factors on the public’s CVI is still unknown [22,23]. Second, the theoretical models in the literature explained only a low level (31.4%) of the variance in Chinese people’s CVI on average (specifically, 18.5% in Lin et al. [24], 49.0% in Fan et al. [25], and 26.6% in Xiao et al. [26]). These models did not include the main factors affecting the public’s CVI, nor did they explain how these factors affect the public’s CVI. Third, although a few studies have developed research models involving trust and perceived benefit (PB) to account for CVI [22,27], the underlying mechanisms of how the indirect effect of trust in vaccines (TV) and PB on CVI is mediated by the public’s attitude toward receiving a COVID-19 vaccination (ARCV) remains unknown. In addition, how TV mediates the relationship between risk perception and CVI has yet to be investigated.

Based on the discussion above, this study aims to develop and test a CVI model explaining the public’s willingness to receive COVID-19 vaccinations by incorporating trust in vaccines, risk perception, and perceived benefit into the theory of planned behavior to understand the factors influencing the public’s willingness to vaccinate. This could contribute to increasing vaccination rates and fill these three research gaps. This study is the first to combine TPB with TV, risk perception, and PB to account for the public’s CVI. Moreover, this study suggests that TV is a mediator in the relationship between risk perception and CVI. This study also proposes that ARCV can mediate the effects of TV and PB on CVI. The findings of this study can provide insights into the interactions between psychological factors, including TV, risk perception, and PB, to determine the public’s CVI. From a practical perspective, the findings can assist policymakers and concerned authorities in adopting effective measures to enhance the public’s CVI. The following section reviews the literature on CVIM factors and presents the model’s development.

## 2. Literature Review and Model Development

### 2.1. Theory of Planned Behavior (TPB)

**Figure 1:**
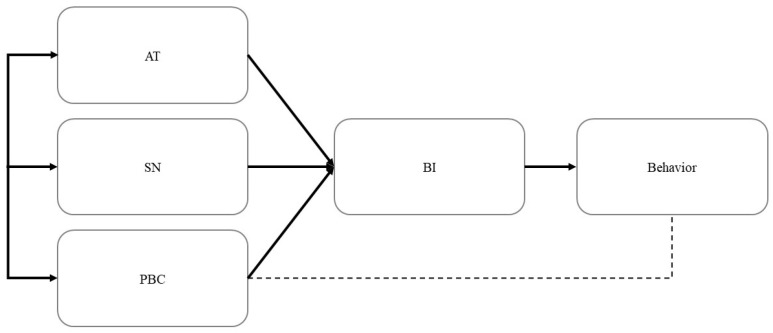
Theory of planned behavior model [37]. Note: AT = attitude; SN = subjective norm; PBC = perceived behavioral control; BI = behavioral intention.

TPB is a theoretical model that proposes linkages between beliefs and behaviors [28]. The theory includes three important positive components, attitude toward a behavior, perceived behavioral control (PBC), and subjective norm (SN), that shape people’s behavioral intentions (Figure 1). The attitude toward a behavior reflects people’s favorable or unfavorable evaluation of a behavior, and PBC is the extent to which people perceive that they can perform a behavior easily. Meanwhile, SN is the extent to which people’s perception of a behavior is affected by the opinions of significant others. Finally, behavioral intention is the extent to which people will perform a behavior. In the research context of this study, ARCV reflects people’s favorable or unfavorable evaluation of receiving a COVID-19 vaccination, SN is the extent to which people’s perception of receiving a COVID-19 vaccination is affected by the opinions of significant others, and PBC is the extent to which people perceive that they can easily receive a COVID-19 vaccination.

TPB is widely used to explain people’s different health-related behaviors, such as tourists’ health risk preventative behavior [29], substance abusers’ safe-sex behavior [30], adolescents’ tobacco smoking behavior [31], construction workers’ use of personal protective equipment [32,33], school girls’ healthy eating habits [34], and the public’s vaccination behavior [35].

TPB has been applied in the context of COVID-19 related behavior. For example, Yahagi et al. [35] used TPB to explain Iranians’ intentions to receive COVID-19 vaccinations. Seong and Hong [36] adopted TPB to elaborate the risk reduction behavior of Koreans during the COVID-19 pandemic. Fan, Chen, Ko, Yen, Lin, Griffiths and Pakpour [25] took TPB into account when explaining the intention of receiving COVID-19 vaccinations among Chinese university students. Overall, the findings of this studies showed that TPB provided remarkable explanatory power in the context of COVID-19-related behavior.

Therefore, for this study, TPB is selected as the theoretical framework for explaining the public’s CVI. Based on TPB, the following hypotheses on ARCV, SN, and PBC are proposed:

**H1.**  *ARCV* *positively influences* *CVI.*

**H2.**  *SN positively influences* *CVI.*

**H3.**  *PBC positively influences* *CVI.*

### 2.2. Trust in Vaccines (TV)

Although numerous and different definitions of trust have been proposed in the literature, a general definition of trust is the voluntary relationship between a trustor and a trustee [38]. In a voluntary relationship, the trustor has expectations on the future actions of the trustee [39]. Trust emerges when vulnerability exists [40]. In a medical context, vulnerability is associated with disease and illness risks and is believed to strengthen trust relationships [40]. In this study, TV is defined as people’s attitude toward the trustworthiness of COVID-19 vaccines. Previous studies have found that trust can positively influence attitudes toward vaccination [41,42]. Trust was also considered to be a positive factor determining the public’s intention to get vaccinated [43]. Based on the above theoretical evidence from previous studies, the following hypotheses are proposed:

**H4.**  *TV positively influences ARCV.*

**H5.**  *TV positively influences CVI.*

### 2.3. Perceived Benefit (PB)

PB describes people’s subjective judgment of the benefits resulting from a behavior, including benefits such as protection and health improvements [44]. In this study, PB includes the benefits that people believe COVID-19 vaccination can provide, including protection against COVID-19 infection, prevention of the spread of COVID-19, and herd immunity [45]. Vaccination is beneficial to not only individuals but also the entire social group [46]. Among Indian people, PB was found to positively influence ARCV [47]. Many studies have integrated TPB and perceived benefit to explain various human behaviors, such as tourists’ health risk preventative behavior [29], bottled water usage [48], vitamin D supplementation intention [49], willingness to cooperate in urban regeneration projects [50], and acceptance of nuclear power plants [51]. These studies theoretically supported the integration of TPB and perceived benefit for explaining the CVI of the public. However, studies have yet to investigate the effect of PB on ARCV among Chinese people. Thus, the following hypothesis is proposed:

**H6.**  *PB positively influences ARCV.*

### 2.4. Risk Perception

Risk perception refers to people’s intuitive risk judgment to evaluate hazards [52]. People are highly concerned about the risks of COVID-19 and COVID-19 vaccination [53,54,55]. Previous studies have demonstrated that the risk perception of a product (from e-government services to automated vehicles) negatively influences trust in the product [56,57]. Additionally, in a study on public compliance with COVID-19 prevention guidelines, risk perception of COVID-19 (RPC) was found to be positively correlated with trust in science [58]. Although there is no consensus on the causality between perceived risk and trust in the literature, the causal influence of perceived risk on trust has been widely demonstrated in human behavioral studies such as consumer behavior [59], technology acceptance [60], travel behavior [61], and the public’s policy compliance during the COVID-19 pandemic [62]. The findings of these studies provided theoretical support for the causal influence of perceived risk on trust in this study. RPC and risk perception of COVID-19 vaccination (RPCV) were found to be related to the public’s CVI [63,64]. Moreover, the findings of Wise et al. [65] and Joslyn et al. [66] suggested that RPC can be used to predict the behavioral intention of the public. However, the relationship between risk perception, TV, and CVI remains unknown [67]. Based on the theoretical evidence on risk perception, the following hypotheses are proposed:

**H7.**  *RPC positively influences TV.*

**H8.**  *RPCV negatively influences TV.*

**H9.**  *RPC positively influences CVI.*

**H10.**  *RPCV negatively influences CVI.*

### 2.5. Model Development

**Figure 2:**
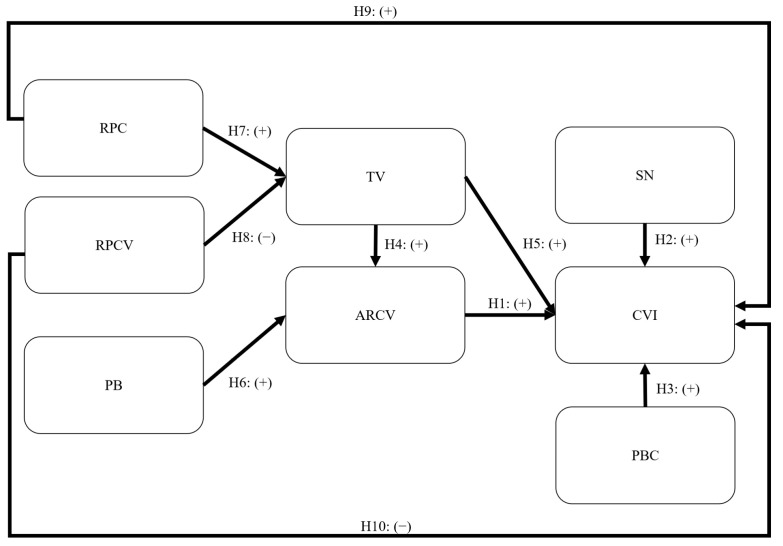
CVIM integrating TV, PB, and risk perception into TPB to explain the public’s CVI. Note: RPC = risk perception of COVID-19; RPCV = risk perception of a COVID-19 vaccination; PB = perceived benefit; TV = trust in vaccines; ARCV = attitude toward receiving a COVID-19 vaccination; SN = subjective norm; CVI = COVID-19 vaccination intention; PBC = perceived behavioral control.

Based on the above literature review, the CVIM is proposed by integrating TV, PB, and risk perception into TPB to explain the public’s CVI. The reason for the use of TPB as the theoretical framework is its high level of effectiveness and parsimony in explaining health-related behaviors [68,69]. In addition, TPB is widely adopted in the context of vaccination [70,71,72]. Figure 2 shows the CVIM with the developed hypotheses. The hypotheses did not indicate the associations between various factors, instead indicating the direction of causality between the variables.

## 3. Methodology

**Figure 3:**
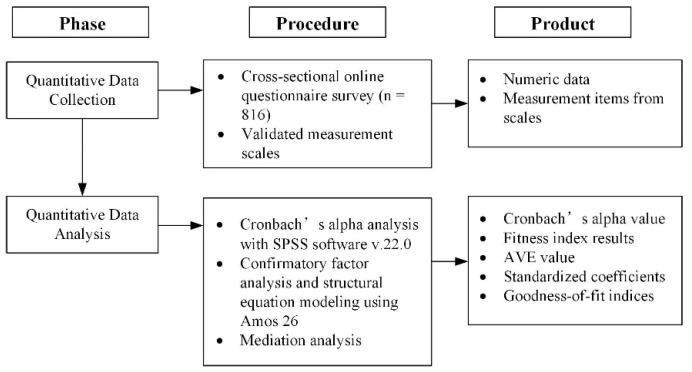
Process of quantitative data collection and analysis.

To investigate the CVIM quantitatively and test the developed hypotheses, a cross-sectional online self-administered survey was used in this study to collect the data. In addition, snowball sampling was conducted to obtain the sample of Chinese adults. This study was conducted from October 2020 to February 2021. Although the COVID-19 vaccination program for emergency use began in July 2020, the target population of the program was high-risk-exposure groups. The high-risk-exposure groups included the staff of quarantine centers/hotels/facilities and designated transport, airport staff, staff of container terminals and shipping services, or cold store practitioners. When the survey took place in October 2020, the COVID-19 vaccination program in China was still not available to the public. This was the rationale for this study to investigate the CVI of the public. At the end of the survey (i.e., February 2021), the public COVID-19 vaccination rate in China was approximately 2.87% (i.e., 40.52 million doses) [73]. The accessibility of vaccination for the public was high in China due to ubiquitous vaccination units, including community health service centers, township health centers, and general hospitals. In the public COVID-19 vaccination program, it was voluntary for the public to receive COVID-19 vaccinations. The study protocol was approved by the Research Committee of the City University of Hong Kong, ethical reference number (MSHSM 20210310) [74]. The participants, measurements, and data analysis are described in the following sections. The process of data collection and analysis is shown in Figure 3.

### 3.1. Participants

The online survey was launched in China, and the study information was shared with the target population (i.e., Chinese adults) via different online social platforms, including Weibo (similar to Twitter), WeChat, and QQ (similar to WhatsApp). These online social platforms were chosen because they are commonly used in China to collect survey data [75]. On the first page of the online survey, the participants were informed of their rights and guaranteed absolute data confidentiality and anonymity. Informed consent was also obtained from each participant. In addition, two inclusion criteria were used to recruit participants: they must be (1) Chinese citizens and (2) at least 18 years old. A total of 838 questionnaires were collected, and after the incomplete questionnaires with missing values were excluded, 816 valid questionnaires were obtained, with an effective rate of 97.4%. The average age of the 816 participants was 30.6 years (SD = 11.4). Table 1 presents the demographic characteristics of the participants. Among the participants, 61.3% were female, 71.4% had at least a bachelor’s degree, and most were employees or freelancers (57.5%). In addition, approximately half (52.5%) of the participants were single.

### 3.2. Measurement

The questionnaire consisted of two parts. The first part was used to gather the participants’ demographic information, including age, gender, education, employment status, and marital status. The second part included items designed and adapted from validated measurement scales in the relevant literature to measure PB, RPC, RPCV, TV, SN, PBC, ARCV, and CVI (Table 2). Specifically, the items related to PBC, ARCV, SN, and CVI were designed based on previous studies that used TPB to explain people’s intentions to get vaccinated [25,35,76]. For RPC and RPCV, the items were developed based on Prasetyo et al. [77], Degarege et al. [78], and Wong [79]. PB was measured using items adapted from Liao et al. [80], while TV was measured with using adapted from Man et al. [81] and Quinn et al. [82]. To ensure that all the items were comprehensible to the participants and suitable to the research context, five experts with more than 10 years of work experience in public health were asked to comment on the clarity and readability of the items. Appropriate modifications were made based on the experts’ comments. All the items were rated by the participants on a seven-point Likert-type scale ranging from 1 (strongly disagree) and 7 (strongly agree). Table 2 presents the details of the measurement items.

### 3.3. Data Analysis

The collected data were analyzed to test and verify the proposed research model (i.e., CVIM). In addition, the important factors affecting CVI were identified. First, SPSS 22 (IBM, New York, NY, USA) was utilized to analyze the internal consistency reliability of the measurements using Cronbach’s alpha. A Cronbach’s alpha value higher than 0.7 demonstrates good internal consistency reliability [83]. Second, Amos 26 was applied to analyze the measurement model and structural model using confirmatory factor analysis (CFA) and structural equation modeling (SEM), respectively. CFA was used to evaluate the construct validity of the measurements (convergent validity and discriminant validity) and to examine the fit between the measurement model and the data. Convergent validity is the extent to which the multiple indicators of a construct are in agreement, whereas discriminant validity is the extent to which constructs differ from one another empirically [84]. Based on the recommendations of Fornell and Larcker [85] for assessing the convergent validity and convergent validity of measurements, the composite reliability (CR) and average variance extracted (AVE) of each construct and factor loadings of the items should be calculated. If the CR values and factor loadings are greater than 0.7 and the AVE value is larger than 0.5, then the convergent validity is considered acceptable. In addition, if the square roots of the AVE of the constructs are greater than the correlations among the constructs, then the discriminant validity is considered acceptable. Third, after CFA was conducted to determine the appropriate measurement model, SEM was utilized to verify whether the proposed research model fits the data and whether the proposed hypotheses were supported. For the CFA and SEM, the *χ*^2^/*df* value, comparative fit index (CFI), Tuck–Lewis index (TLI), and root mean square error of approximation (RMSEA) were used to evaluate the statistical fitting of the measurement and structural models [83]. When the TLI and CFI are higher than 0.95, the *χ*^2^/*df* value is less than 5, and the RMSEA is less than 0.08, the model has an acceptable fit [84]. Finally, a mediation analysis was conducted to examine the mediating role of TV in the relationship between RPC, RPCV, ARCV, and CVI. The extent to which ARCV mediates the relationship between PB and CVI was also examined. In the mediation analysis, the *p*-value program developed by Falk and Biesanz [86] was used to make inferences about the indirect effect. The *p*-value program is widely used in research on human behavior [87,88,89].

## 4. Results

### 4.1. Measurement Model Assessment

To improve the fit of the measurement model with the data, the measurement model was modified by deleting four items with factor loadings smaller than 0.7. Specifically, one item (PBC1) was deleted from the PBC construct, two items (ARCV1 and ARCV3) were deleted from the ARCV construct, and one item (RPC3) was deleted from the RPC construct. The fitness index results of the modified model are shown in Table 3. The *χ*^2^/*df* value of the modified measurement model was 3.315, which is less than 5. The TLI and CFI values were greater than 0.95, and the RMSEA value was less than 0.08. The results reflected the good fit between the measurement model and the data. As demonstrated in Table 4, all the AVE values were greater than 0.5, which is the minimum acceptable value. Moreover, all the CR values and factor loadings were greater than the minimum required level of 0.7. Therefore, it can be concluded that the measurements demonstrated good convergent validity. In addition, the measurements exhibited good internal consistency reliability, which was reflected by the Cronbach’s alpha values of all the constructs, which were greater than 0.7. Furthermore, the diagonal and off-diagonal values in Table 5 represent the square roots of the AVE of the constructs and correlations between two constructs, respectively. As each square root of the AVE was larger than any binary correlation, it can be concluded that the measurements demonstrated good discriminant validity.

### 4.2. Structural Model Assessment

**Figure 4:**
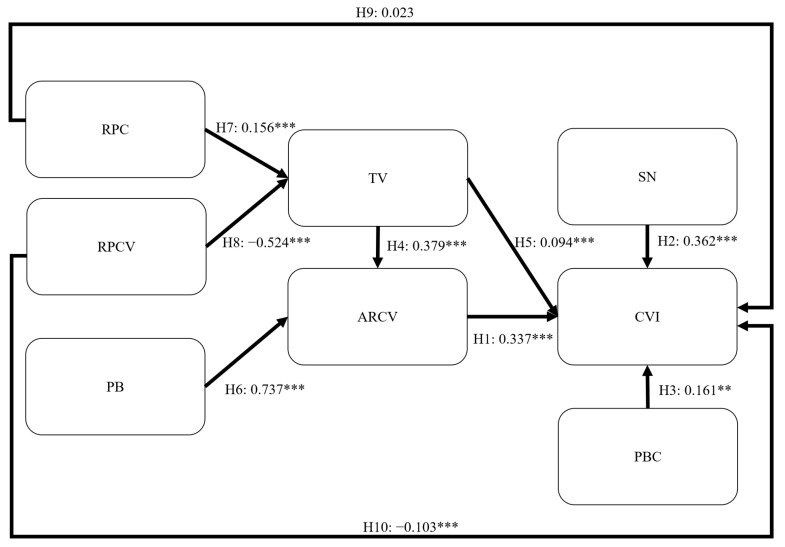
Structural model assessment results; *** *p* < 0.001 and ** *p* < 0.01. Note: RPC = risk perception of COVID-19; RPCV = risk perception of a COVID-19 vaccination; PB = perceived benefit; TV = trust in vaccines; ARCV = attitude toward receiving a COVID-19 vaccination; SN = subjective norm; CVI = COVID-19 vaccination intention; PBC = perceived behavioral control.

The results showed that all the goodness-of-fit indices of the structural model satisfied the recommended criteria (Table 3), demonstrating that the CVIM can appropriately represent the hypothesized relationships between the constructs. Figure 4 presents the results of the structural model assessment. Table 6 illustrates the hypothesis testing results. The three hypotheses on TPB were supported. Specifically, ARCV (H1; *β* = 0.337, *p* < 0.001), SN (H2; *β* = 0.362, *p* < 0.001), and PBC (H3; *β* = 0.161, *p* < 0.01) had a significant and positive impact on CVI. In addition, TV (H5; *β* = 0.094, *p* < 0.001) had a significant and positive effect on CVI. The results also demonstrated that TV (H4; *β* = 0.379, *p* < 0.001) and PB (H6; *β* = 0.737, *p* < 0.001) had a significant and positive impact on ARCV. Furthermore, RPC (H7; *β* = 0.156, *p* < 0.001) significantly and positively influenced TV, whereas RPCV (H8; *β* = −0.524, *p* < 0.001) significantly and negatively influenced TV. RPC (H9; *β* = 0.023, *p* = 0.204) did not significantly positively influence CVI, whereas RPCV (H10; *β* = −0.103, *p* < 0.001) significantly and negatively influenced CVI. The CVIM explained 81.99% of the variance in ARCV and 32.0% of the variance in TV. In addition, the CVIM explained 80.3% of the variance in CVI.

### 4.3. Mediation Analysis

Table 7 summarizes the results of the mediation analysis. TV served as an important meditator in the relationship between RPC, RPCV, ARCV, and CVI. As mediated by TV, the indirect effect of RPC on ARCV and CVI was significant and positive, that is, 0.06 (*p* < 0.001) and 0.02 (*p* < 0.001), respectively. The indirect effect of RPCV on ARCV and CVI was significant and negative, that is, −0.20 (*p* < 0.001) and −0.08 (*p* < 0.001), respectively.

ARCV was found to be a significant mediator in the relationships between PB and CVI and between TV and CVI. The indirect effect of PB and TV on CVI mediated by ARCV was significant and positive, that is, 0.26 (*p* < 0.001) and 0.13 (*p* < 0.001), respectively.

## 5. Discussion

This study developed the CVIM by extending TPB with TV, risk perception, and PB. The CVIM showed that people’s willingness to receive COVID-19 vaccinations was directly related to their TV, ARCV, SN, and PBC. In addition, PB and TV were found to influence ARCV directly, whereas risk perception was found to influence TV directly. The results provided supporting evidence for the CVIM. Thus, the findings of this study can contribute to relevant research and practice.

### 5.1. Theoretical Implications

Although previous studies have used TPB to explain people’s CVI, this study is the first to incorporate TV, risk perception, and PB into TPB to explain this phenomenon. Consistent with the findings of previous studies, those of this study supported the hypotheses related to TPB. Specifically, ARCV, SN, and PBC were found to positively influence CVI. These results successfully validated the theoretical contribution of TPB in understanding the CVI of Chinese people. In addition, ARCV and SN were found to influence CVI more than PBC, which agrees with the findings of Caso, Capasso, Fabbricatore, and Conner [70]. The theoretical models in the literature can explain only approximately 31.4% of the variance in Chinese people’s CVI, on average (specifically, 18.5% in Lin, Hu, Zhao, Alias, Danaee, and Wong [24], 49.0% in Fan, Chen, Ko, Yen, Lin, Griffiths, and Pakpour [25], and 26.6% in Xiao, Liu, Wang, Mao, Chen, Li, Liu, Dai, Gao, and Fu [26]). Compared with the theoretical models, the CVIM demonstrated better explanatory power, accounting for 78.8% of the variance in CVI.

This study determined that TV was a significant and positive predictor of CVI. This finding is similar to that of Ahorsu, Lin, Yahaghai, Alimoradi, Broström, Griffiths, and Pakpour [43], who observed the positive influence of trust in the healthcare system on people’s willingness to receive COVID-19 vaccinations. In addition, TV was found to have a significant and positive indirect effect on CVI, mediated by ARCV. Trust is considered as a tool for reducing cognitive complexity and facilitating the decision-making process [90]. When people encounter uncertain situations and are requested to take action, trust may serve as a key to specific risk problems [56]. This study suggested that when people decide to receive a COVID-19 vaccination, their TV will play a critical role. These findings are unique and significant to the literature, as studies have yet to examine how TV influences CVI.

This study is the first to consider two types of risk perception to explain the public’s CVI, namely, RPC and RPCV. The result showed that RPC positively and indirectly influenced CVI through the mediation of TV. The public who perceived a high risk of COVID-19 tended to trust vaccines and thus intended to receive COVID-19 vaccinations. This result supported the important meditating role of TV in the relationship between RPC and CVI and contributed to our understanding of how RPC affects the CVI of the public. The results demonstrated that both types of risk perception were significant factors determining TV, similar to the findings of Mutimukwe, Kolkowska, and Grönlund [57] and Plohl and Musil [58]. Mutimukwe, Kolkowska, and Grönlund [57] found that risk perception of a product negatively influences trust in the product, whereas Plohl and Musil [58] reported that RPC is positively correlated with trust in science [58]. This study showed that RPC did not affect CVI among Chinese adults, but RPCV did. This is inconsistent with the findings of Wise, Zbozinek, Michelini, Hagan, and Mobbs [65] and Joslyn, Savelli, Duarte, Burgeno, Qin, Han, and Gulacsik [66], who demonstrated that RPC can be used to predict the behavioral intention of the public. One possible reason for this inconsistency is the underestimation of the risks of the COVID-19 pandemic among Chinese adults [91]. Such underestimation may be due to the small number of COVID-19-related deaths in China [92]. On the other hand, this study demonstrated that RPCV negatively influences CVI among Chinese adults, which is consistent with the findings of Zheng, Jiang, and Wu [64]. Zheng, Jiang, and Wu [64] found that perceived susceptibility to the side effects of COVID-19 vaccines negatively influences vaccination intention among Americans. This study affirmed the important role of RPCV in explaining CVI among Chinese adults.

The findings of this study also successfully demonstrated the underlying mechanisms of risk perception in shaping CVI and ARCV. Specifically, the indirect effect of both types of risk perception on CVI and ARCV was significant and mediated by TV. However, the indirect effect of RPC on CVI and ARCV was positive, whereas the effect of RPCV was negative. When people perceive risks associated with COVID-19 vaccinations, their trust in COVID-19 vaccines will be reduced, and their willingness to receive vaccinations will decrease. By contrast, when people perceive COVID-19 to be life threatening, their TV will increase, and they will want to be vaccinated. The rapid development of COVID-19 vaccines caused public concern about their safety [93]. Most Chinese people believe that COVID-19 is a serious disease but underestimate their risk of contracting the virus [94]. As a result, they may hesitate to get vaccinated. The findings of this study contribute to the relevant literature on public health by providing an understanding of how the two types of risk perception influence CVI and ARCV.

This study indicated that PB positively influenced ARCV, which is consistent with the finding of Mir, Parveen, Mullick, and Nabi [47], who found that PB positively influences ARCV among Indian people. When people perceive that COVID-19 vaccination can protect them against COVID-19 infection, prevent the spread of COVID-19, and achieve herd immunity, they tend to have a positive ARCV. Although PB was found to be a positive predictor of willingness to get vaccinated [95], the underlying mechanism of how PB influenced CVI has not been well-examined in the literature. However, this study successfully addressed this research issue by showing that the indirect effect of PB on CVI was significant, positive, and mediated by ARCV, thereby confirming the important role of PB in determining CVI.

### 5.2. Practical Implications

The findings of this study can help governments and vaccine developers establish effective interventions to promote vaccination against COVID-19. Based on the findings, several practical recommendations are proposed. First, RPCV had a negative direct effect on TV and a negative indirect effect on CVI. In addition, RPC had a positive direct effect on TV and a positive indirect effect on CVI. Hence, the safety and transparency of COVID-19 vaccines should be clearly explained by relevant authorities and vaccine developers to the public to reduce the perceived risk related to COVID-19 vaccinations [96]. In the recent COVID-19 vaccination work program, China’s National Health and Wellness Commission indicated the need for continued emphasis on vaccination safety, focusing on the purpose and significance of vaccination, and actively guiding the target population to take the vaccine initiative [97]. Furthermore, information on the adverse effects of COVID-19 should be widely disseminated to the public using different social media platforms to increase the perceived risk related to the virus [98], thereby increasing the public’s TV and CVI.

Second, PB had a positive direct effect on ARCV and a positive indirect effect on CVI. Therefore, the efficacy of COVID-19 vaccines in reducing the risk of contracting COVID-19 should be highlighted in COVID-19 vaccination programs so the public can clearly understand the potential benefits of receiving a COVID-19 vaccination [96]. The Hong Kong Health Protection Centre promoted the COVID-19 vaccine through its website and has developed a COVID-19 vaccination program to provide the public with answers to questions about the vaccine and vaccination appointment services [99]. In addition, the U.S. Centers for Disease Control and Prevention also published information on the use of the COVID-19 vaccine on its website, including the appropriate population for vaccination and the appropriate age for vaccination [100]. When people perceive that receiving a COVID-19 vaccination is beneficial, their ARCV will be positive, thereby enhancing their CVI.

Finally, establishing and improving vaccine development supervision and standardizing vaccination services are the key to promoting COVID-19 vaccination [101]. In the event of serious adverse reactions after a COVID-19 vaccination, prompt targeted treatments or measures should be provided to increase the public’s TV and thus CVI. In addition, we can also extract effective methods for COVID-19 vaccination from similar promotion techniques for mumps, measles, and rubella vaccines for reference [102,103]. For example, providing preemptive education to medical staff, providing detailed information on various complaints such as COVID-19 vaccination and its complications, and understanding the negative effects of different types of COVID-19 vaccination, are necessary in order to better improve the vaccine. In addition, we can learn effective methods for COVID-19 vaccination from similar promotion techniques for mumps, measles, and rubella vaccines [102,103]. Effective measurements include offering preemptive education to healthcare workers, providing detailed information on various complaints such as COVID-19 vaccination and its complications, and illustrating the negative effects of different types of COVID-19 vaccinations. The government can also incorporate the latest evidence on the efficacy of the COVID-19 vaccine and the risk of morbidity and mortality, thus moderately reducing the unknowns and fears of the public about vaccination [104].

### 5.3. Limitations and Directions for Future Research

This study has several limitations, which can guide future research. First, the study participants were all from China, limiting the generalizability of the findings. Future research may involve conducting this study in other countries and cultures to further validate the CVIM and gain a more complete understanding of the public’s CVI. Also, it may be beneficial to conduct broader cross-cultural and international research to understand the public’s CVI comprehensively. Second, this study excluded young people aged below 18. Considering the increasing vaccination behavior targeting young people, substantial future research should be devoted to examining the generalizability of the findings to young people and understanding their CVI. Third, this study focused on CVI but not on the public’s actual behavior. The association between behavioral intentions and actual behaviors in the context of this study should be extensively studied in order to gain a more comprehensive understanding of the actual situation of COVID-19 vaccination in the future. Fourth, this study did not collect data about the vaccination status of the participants. The vaccination status of the participants may be the key predictor of the COVID-19 vaccination intention. Future research should consider the vaccination status of the public in predicting their vaccination intention. Also, the target behavior would not be the same for vaccinated and non-vaccinated participants. The underlying background factors of vaccination intention may strongly differ between vaccinated and non-vaccinated individuals. This research area should be investigated by conducting a multi-group analysis (between vaccinated and non-vaccinated people) in the future.

## 6. Conclusions

This study proposed and tested the CVIM to explain the public’s CVI. The CVIM was developed by incorporating TV, PB, and risk perception into TPB. Theoretically, the results demonstrated the applicability of TPB to the research context of CVI. In addition, the important role of TV, PB, and risk perception in determining CVI was confirmed. Compared with previous research models that explained approximately 31.4% of the variance in Chinese people’s CVI, on average, the developed and validated CVIM had a stronger explanatory power, accounting for 78.8% of the variance in CVI. Moreover, the underlying mechanism of how risk perception and PBC influenced CVI, with TV and ARCV as mediators, was determined. Pragmatically, the findings are expected to benefit governments and vaccine developers in establishing effective interventions to promote vaccination against COVID-19. Furthermore, several practical recommendations are provided to improve COVID-19 vaccination rates.

## Figures and Tables

**Table 1:** Demographic characteristics of participants.

Characteristics	Frequency	Percentage (%)
Gender		
Male	316	38.7
Female	500	61.3
Education		
Grade 12 or lower	26	3.2
High school graduate	207	25.4
Bachelor’s degree	474	58.1
Postgraduate degree	109	13.3
Employment status		
Student	338	41.4
Full-time employee	218	26.7
Part-time employee	119	14.6
Freelancer	132	16.2
Retired	4	0.5
Unemployed	5	0.6
Marital status		
Married	339	41.5
Single	428	52.5
Divorced or widowed	49	6.0

**Table 2:** Measurement items.

Construct	Item	Content
PB	PB1	I believe that receiving a COVID-19 vaccination can protect me against COVID-19.
	PB2	I believe that receiving a COVID-19 vaccination can help protect my family and friends against COVID-19.
	PB3	I believe that receiving a COVID-19 vaccination can reduce my risk of contracting COVID-19.
PBC	PBC1	I can decide for myself whether or not to receive a COVID-19 vaccination.
	PBC2	I can decide on my own when to receive a COVID-19 vaccination.
	PBC3	I can decide where to receive a COVID-19 vaccination.
	PBC4	I can afford the price of a COVID-19 vaccination.
SN	SN1	My friends think I should receive a COVID-19 vaccination.
	SN2	My family thinks I should receive a COVID-19 vaccination.
	SN3	The person most important to me think I should receive a COVID-19 vaccination.
RPCV	RPCV1	I am worried that a COVID-19 vaccination may cause facial palsy or other serious movement disorders such as paralysis.
	RPCV2	I am worried that a COVID-19 vaccination may cause infection of other serious diseases.
	RPCV3	I am worried about being infected by other people in the process of getting a vaccination.
	RPCV4	I am worried that the mutation of COVID-19 will make the vaccines ineffective.
RPC	RPC1	I think that COVID-19 is a serious disease.
	RPC2	I think that COVID-19 can lead to death.
	RPC3	I think that COVID-19 can affect mental health.
	RPC4	I think the COVID-19 pandemic will continue indefinitely.
	RPC5	I think I am very vulnerable to COVID-19.
	RPC6	I think COVID-19 mutates easily.
ARCV	ARCV1	Receiving a COVID-19 vaccination is a good idea.
	ARCV2	Receiving a COVID-19 vaccination is a wise idea.
	ARCV3	Receiving a COVID-19 vaccination is pleasant.
	ARCV4	Receiving a COVID-19 vaccination is necessary.
	ARCV5	Receiving a COVID-19 vaccination is acceptable.
TV	TV1	COVID-19 vaccines are dependable.
	TV2	COVID-19 vaccines are reliable.
	TV3	Overall, I can trust COVID-19 vaccines.
CVI	CVI1	I want to receive a COVID-19 vaccination.
	CVI2	I predict that I will receive a COVID-19 vaccination in the future.
	CVI3	I plan to receive a COVID-19 vaccination in the future.

Note: RPC = risk perception of COVID-19; RPCV = risk perception of a COVID-19 vaccination; PB = perceived benefit; TV = trust in vaccines; ARCV = attitude toward receiving a COVID-19 vaccination; SN = subjective norm; CVI = COVID-19 vaccination intention; PBC = perceived behavioral control.

**Table 3:** Model fit indices for testing the measurement and structural models.

Model Fit Index	Recommended Value	Measurement Model	Structural Model
*χ*^2^/*df*	5	3.315	4.831
CFI	0.95	0.979	0.964
TLI	0.95	0.975	0.959
RMSEA	0.08	0.053	0.069

**Table 4:** CFA results, convergence validity, and internal consistency reliability.

Construct	Item	Factor Loading	AVE	CR	Cronbach’s Alpha
PB	PB1	0.875	0.831	0.937	0.936
	PB2	0.932			
	PB3	0.927			
PBC	PBC2	0.930	0.847	0.943	0.940
	PBC3	0.946			
	PBC4	0.884			
SN	SN1	0.910	0.870	0.953	0.952
	SN2	0.949			
	SN3	0.939			
RPCV	RPCV1	0.995	0.960	0.990	0.990
	RPCV2	0.961			
	RPCV3	0.995			
	RPCV4	0.968			
RPC	RPC1	0.823	0.751	0.938	0.937
	RPC2	0.889			
	RPC4	0.928			
	RPC5	0.828			
	RPC6	0.861			
ARCV	ATT2	0.885	0.808	0.926	0.925
	ATT4	0.874			
	ATT5	0.936			
TV	TV1	0.935	0.879	0.956	0.955
	TV2	0.957			
	TV3	0.920			
CVI	CVI1	0.961	0.946	0.981	0.981
	CVI2	0.984			
	CVI3	0.973			

Note: RPC = risk perception of COVID-19; RPCV = risk perception of a COVID-19 vaccination; PB = perceived benefit; TV = trust in vaccines; ARCV = attitude toward receiving a COVID-19 vaccination; SN = subjective norm; CVI = COVID-19 vaccination intention; PBC = perceived behavioral control.

**Table 5:** Discriminant validity assessment results.

	RPC	RPCV	TV	ARCV	SN	PBC	PB	CVI
RPC	**0.867**							
RPCV	−0.130	**0.980**						
TV	0.216	−0.545	**0.938**					
ARCV	0.264	−0.436	0.776	**0.899**				
SN	0.247	−0.366	0.653	0.878	**0.933**			
PBC	0.204	−0.357	0.629	0.860	0.899	**0.920**		
PB	0.215	−0.381	0.657	0.860	0.821	0.867	**0.912**	
CVI	0.254	−0.475	0.725	0.875	0.861	0.843	0.829	**0.973**

Note: The square roots of the AVE values are shown as the diagonal values in bold. RPC = risk perception of COVID-19; RPCV = risk perception of a COVID-19 vaccination; PB = perceived benefit; TV = trust in vaccines; ARCV = attitude toward receiving a COVID-19 vaccination; SN = subjective norm; CVI = COVID-19 vaccination intention; PBC = perceived behavioral control.

**Table 6:** Hypothesis testing results.

Hypothesis	Standardized Path Coefficient	*p*-Value	Result
H1: ARCV → CVI	0.349	<0.001	Supported
H2: SN → CVI	0.379	<0.001	Supported
H3: PBC → CVI	0.158	<0.01	Supported
H4: TV → ARCV	0.378	<0.001	Supported
H5: TV → CVI	0.154	<0.001	Supported
H6: PB → ARCV	0.737	<0.001	Supported
H7: RPC → TV	0.157	<0.001	Supported
H8: RPCV → TV	–0.527	<0.001	Supported

Note: RPC = risk perception of COVID-19; RPCV = risk perception of a COVID-19 vaccination; PB = perceived benefit; TV = trust in vaccines; ARCV = attitude toward receiving a COVID-19 vaccination; SN = subjective norm; CVI = COVID-19 vaccination intention; PBC = perceived behavioral control.

**Table 7:** Mediation analysis results.

Independent Variable	Mediator	Dependent Variable	Standardized Indirect Effect	*p*-Value	Result
RPC	TV	ARCV	0.06	<0.001	Significant
RPCV	TV	ARCV	−0.20	<0.001	Significant
RPC	TV	CVI	0.01	<0.001	Significant
RPCV	TV	CVI	−0.05	<0.001	Significant
PB	ARCV	CVI	0.25	<0.001	Significant
TV	ARCV	CVI	0.13	<0.001	Significant

Note: RPC = risk perception of COVID-19; RPCV = risk perception of a COVID-19 vaccination; PB = perceived benefit; TV = trust in vaccines; ARCV = attitude toward receiving a COVID-19 vaccination; SN = subjective norm; CVI = COVID-19 vaccination intention.

## Data Availability

The data presented in this study are available on request from the corresponding author.
